# Academic global health collaboration: the Ruhuna-Duke partnership

**DOI:** 10.1080/16549716.2025.2543603

**Published:** 2025-08-21

**Authors:** Champica K Bodinayake, Ajith De S Nagahawatte, Vijitha de Silva, P L Ariyananda, Bilesha Perera, Ruvini Kurukulasooriya, M H Madureka Premamali, Christopher W Woods, L Gayani Tillekeratne, Truls Østbye

**Affiliations:** a Department of Medicine, Faculty of Medicine, University of Ruhuna, Galle, Sri Lanka; b Duke Global Health Institute, Duke University, Durham, NC, USA; c Duke-Ruhuna Collaborative Research Centre, Faculty of Medicine, University of Ruhuna, Galle, Sri Lanka; d Professorial Unit of Medicine, Teaching Hospital Karapitiya/National Hospital Galle, Galle, Sri Lanka; e Duke Hubert-Yeargan Center for Global Health, Duke University, Durham, NC, USA; f Department of Microbiology, Faculty of Medicine, University of Ruhuna, Galle, Sri Lanka; g Department of Community Medicine, Faculty of Medicine, University of Ruhuna, Galle, Sri Lanka; h Department of Medicine, Duke University School of Medicine, Durham, NC, USA; i Department of Family Medicine and Community Health, Duke University School of Medicine, Durham, NC, USA

**Keywords:** Capacity building, collaboration between universities, education & research, global health, Sri Lanka

## Abstract

The role of institutional partnerships is increasingly recognized as a means of advancing our collective efficacy in improving public health. Shared challenges related to infectious and chronic diseases, as well as social determinants of health including environmental stressors, have led to a growth in academic global health collaborations. Triggered by the 2004 tsunami, the University of Ruhuna, Sri Lanka, and Duke University, USA, established an educational and research collaboration that has been sustained and broadened over two decades. The initiation and development of the collaboration, as well as its educational and research components, are described in this manuscript. We discuss lessons learned that may be of interest to other emerging partnerships: the keys to the collaboration’s success, challenges and barriers faced, as well as plans to sustain and further grow an equitable, academically rigorous, and impactful global health partnership.

## Paper Context

- **Main findings**: The initiation and development of the Duke-Ruhuna collaboration, as well as its educational and research components, are described, and lessons learned are discussed.
- **Added knowledge**: The breadth of educational and research activities and the strength of the relationship between partners in Sri Lanka and the US have helped sustain the Ruhuna-Duke academic collaboration over two decades, overcoming the many challenges encountered. The collaboration owes its success to the commitment, initiative, and dedication from faculty, students, and staff, and not least to the transparency and mutual trust that has increasingly been built and established.
- **Global health impact for policy and action**: The experience and lessons learned from the challenges and barriers faced, as well as plans to sustain and further grow an equitable, academically rigorous, and impactful global health partnership provide lessons for other emerging global health partnerships.

## Background

### Initiation and development

The early years of this century saw a strong drive towards academic internationalization due to growing concern about infectious and chronic disease challenges and increasing recognition of the importance of social determinants of health. This drive has led to substantial growth in collaborative ‘global health’ projects and linkages of universities in North America and Europe with partners in the ‘Global South.’

Recent reports from global health partnerships and collaborations [1–6] note their promise for strengthening institutional capacity in low- or middle-income countries. Earlier partnerships have been organized around specific public health contents (such as HIV/AIDS or food security) or skill areas (such as research or workforce development). The relatively small number of examples in the literature highlight the importance of sharing experiences. A collaborative impact model is a framework that encourages individual organizations to form a network to work together to achieve community-wide outcomes. It requires several elements such as a common agenda, shared measurement systems, activities that reinforce programs across the network, continuous communication, and a backbone support organization to drive the overall initiative to success [7]. In this article, we outline the establishment of the Ruhuna-Duke collaboration in the global health context. Further, we describe two decades of changes, growth, and present key lessons learned, emphasizing aspects of the collaborative impact model that we followed, in order to build a successful collaboration.

The collaboration started immediately after the Great 2004 Tsunami in the Indian Ocean, which heavily impacted southern Sri Lanka [8]. The Duke University Health System Chancellor sponsored the collection of funds (~75,000 USD) to support a response to the disaster. However, given the large number of aid organizations in place and the fact that the local health system responded well to the tsunami, the funds were designated to establish a long-term collaboration with a partner academic institution. University of Ruhuna was chosen given that the southern coast nearby had been devastated by the tsunami. A Memorandum of Understanding (MOU) was signed in 2006 between Duke and Ruhuna Universities and renewed in 2010 as an indefinite agreement with the objectives of strengthening and facilitating ongoing and new research activities, enabling student/resident exchange training programs, and exploring opportunities for collaborating with other national and international universities.

The initial Duke collaborators represented the Departments of Internal Medicine and Family Medicine, with administrative support from the Duke Hubert-Yeargan Center for Global Health (HYC). The Collaboration has since expanded to include a broad range of Duke representatives including Pathology, Psychiatry, Nursing, the Physician Assistant Programme, and the School of Environmental Health. When the Duke Global Health Institute (DGHI) was established in 2006, this became the administrative home for the US side of the collaboration.

The University of Ruhuna (mainly the Faculty of Medicine) became the primary home of the collaboration on the Sri Lankan side (The Duke-Ruhuna Collaborative Research Centre, https://dukeruhuna.org/) [9], later joined by the Faculties of Allied Health Sciences, Science, and Humanities and Social Sciences. Enthusiastic support from the Ruhuna and Duke leadership drove the initial establishment and later expansion, including partnerships with Duke Kunshan University (China) and Duke-National University of Singapore (Duke-NUS). Later, collaboration extended to Duke’s other partnership locations in South America, Africa, and elsewhere in Asia and now involves several other Sri Lankan universities (Jaffna, Kelaniya, Peradeniya, and General Sir John Kotelawala Defence University), Fudan University (China), and the University of Amsterdam (the Netherlands).

The population health research around Galle involved collaboration with the Sri Lanka Ministry of Health (the Epidemiology Unit, public health inspectors, and public health midwives). The clinical research primarily took place at the affiliated public hospital, Teaching Hospital Karapitiya (THK, renamed as National Hospital Galle in August 2024), an 1800-bed tertiary hospital, with recent expansion to over 15 other hospitals. Private medical laboratories in Sri Lanka (Gene Labs, Genetech, and Durdans) and collaborators from the University of North Carolina in Chapel Hill, Johns Hopkins University, and the Centers for Disease Control and Prevention in the US have also supported the efforts. Continuous and clear communication was essential for the development of the collaboration, and was conducted through regular partnership meetings, email communications, internal reports and planning documents, collaborative planning sessions, and reflective discussions among team members. The descriptive analysis presented in this manuscript is based on an internal review and agreement among key participants at both institutions.

## Research

The very first project was prompted by the Sri Lankan colleagues and included assessing medical records at THK pre- and post-tsunami to understand changes in disease epidemiology [8]. Although the research portfolio of the collaboration is now broad and eclectic, three interrelated areas of research have emerged with time: infectious disease, community medicine (especially occupational health/health of vulnerable workers), and mental health across the life span.

### Infectious disease-related research

The research activities in infectious diseases grew out of a large, prospective, surveillance study of >1000 patients to determine the etiology of community-onset fever in patients at THK. Laboratory-based, gold-standard testing was performed to identify the prevalence of pathogens such as *Leptospira* spp., *Rickettsia* spp., and dengue [10–12]. This initial work was followed by several other large surveillance studies to understand the changing epidemiology of febrile and respiratory illnesses in the inpatient and outpatient settings. These studies have since become more focused to explore specific pathogens (e.g. dengue or influenza) and to explore clinical questions such as predictors of severity [13–29]. While laboratory testing was initially conducted at Duke’s facilities in North Carolina and Singapore, over time, both physical and human resource capacity at the Department of Microbiology, Ruhuna, developed such that advanced molecular testing as well as basic microbiologic testing was supported on site. This infrastructure was utilized to conduct testing for an outbreak of adenovirus, respiratory syncytial virus, and influenza in southern Sri Lanka in 2018 and was later used to support the national response to the SARS-CoV-2 pandemic. The data generated were shared real-time with the Ministry of Health. More recently, the infectious disease program has shifted to interventions for improving appropriate antibiotic use and decreasing multidrug-resistant pathogens such as extended spectrum beta-lactamase (ESBL)-producing organisms, carbapenem-resistant bacteria, and methicillin-resistant *Staphylococcus aureus* (MRSA) [30–39].

In addition, exploring connections between humans, animals, and the environment to understand the impact on health under the One Health model has now become an important part of the work.

Over time, funding for these studies has shifted from mostly US sources (National Institutes of Health, Department of Defense, DGHI, and Duke HYC) to a combination of US and Sri Lankan sources (National Science Foundation, National Research Council, University Grants Commission). Similarly, data management and analysis are now shared between the two sites.

In 2020, the Ruhuna-Duke Centre for Infectious Diseases (RDCID) was approved as a formal center within the university by the Senate of the University of Ruhuna (https://rdcid.dukeruhuna.org/) [40]. This centre was formed to consolidate the work in infectious diseases over the past two decades and to continue the expansion of research and training opportunities locally and regionally.

### Community-based research

Complementing and frequently overlapping with the infectious disease projects above, the collaboration has also had a community health focus, aiming to understand the epidemiology of diseases and risk factors in the community, especially among vulnerable groups. The aim of these studies was to address important public health issues and engage with local communities to ultimately improve health care and improve health status. Students and faculty work with community members to identify research priorities that are relevant to the local population’s health needs. These priorities inform the focus of the collaboration’s broad and eclectic range of research projects.

Over and beyond the infectious disease projects mentioned above, the community-oriented projects include acute and chronic disease prevention [41,42] and epidemiology, maternal [43,44], child health [30], health of the elderly and their caregivers [45–48], road safety and under-reporting of road traffic injuries in Sri Lanka [49–51], substance use [52,53], violence among adolescents [54–56], and mental health across the lifespan [57–66]. A strong focus throughout the collaboration has been occupational health, to a large extent driven by the interests and expertise of the Ruhuna research faculty. The latter include systematic reviews [67], quantitative/epidemiological and qualitative studies of a wide range of vulnerable workers, including textile workers in the free trade zone [68–70], foreign domestic workers returning from the Middle East [71], rubber plantation workers [72–74], nurses, tuk-tuk (three-wheel taxi) drivers [53,75], farm workers, public health midwives [76], and construction workers.

## Education and training

While the initial focus was research, education and training of both Duke and Ruhuna learners early became and still remains a central and integral part of the collaboration.

### Research student projects

The most substantial educational component has combined research and education. Around 30 students have completed research projects as part of their graduate studies (MSc Global Health from Duke in Durham and from Duke Kunshan or medical students from Duke or from Duke-NUS). In addition, the collaboration has sponsored students from other universities such as Johns Hopkins University and North Carolina State University. The projects are based on an important local public health problem and align with the research interests of Ruhuna and Duke researchers outlined above. Publication in a public health or medical journal has been strongly encouraged from the start of each project, and the results from the majority of the projects have been published [13,16,21,30,31,34,35,37–39,53,69,77,78] and shared with local stakeholders. Project funding provided to the students covers travel, accommodation, and a local research assistant/translator, who plays a key role in the success of the project and often a graduate student from Ruhuna, who can concurrently use data from the same project for their Master’s thesis from Ruhuna, resulting in ***‘twinning projects’*** that lead to at least two publications with shared authorship [71–74]. While most research students/trainees are from Duke’s main campus, participation from Duke-NUS, Duke Kunshan, and Ruhuna is growing. For example, the local onsite coordinator completed a PhD in Molecular Microbiology under joint Ruhuna-Duke supervision, inspiring a staff development model [33,34]. In addition, over 70 research assistants (often MBBS pre-interns) have gained research skills, and numerous Ruhuna faculty have developed expertise in research methodology, analysis, and academic writing.

### Clinical electives for medical students and residents

University of Ruhuna has a well-established ‘Clinical Elective for Foreign Medical Students,’ allowing medical students or Physician Assistant students from Duke and Duke NUS to choose rotations in surgery, pediatrics, and community medicine. The Duke students usually pay a tuition fee of 75 USD per week to Ruhuna for this rotation [79]. Furthermore, Duke medical residents (organized by the Duke Hubert-Yeargan Center for Global Health) participate in 8–10-week electives in internal medicine, pediatrics, psychiatry, and family medicine at THK, gaining valuable concrete hands-on experience in a diverse and challenging resource-limited clinical environment.

### Undergraduate global health course

In collaboration with the University of Amsterdam, ‘Global Health in Context: Sri Lanka,’ a 4-week group-based course combining experiential learning in the field with small group projects, was successfully implemented. Each group included Duke students working with medical students from the Netherlands, thus adding an additional global dimension to their learning.

### Research methodology and statistics training

The Duke HYC has also conducted a 6-month virtual courses and webinar series related to research methodology and statistics, geared towards junior faculty and postgraduate students at Ruhuna and other international partnerships. A total of 22 Ruhuna faculty/postgraduates have completed this course over the years.

### MDs specialty overseas training

The collaboration has also provided opportunities for Sri Lankan physicians who need to complete at least a year of overseas training as part of their specialty (MD) training, funded by the Sri Lanka Ministry of Health, to gain overseas training at Duke. They have followed structured research methods courses, engaged in partner research projects, and produced manuscripts [22,36,46,80,81]. Upon returning to Sri Lanka, these physicians often attain senior roles in the Sri Lankan academic and health system, facilitate further collaborative research projects, and co-mentor research students.

## Discussion: lessons learned

### Support from relevant stakeholders

The positive culture of partnership between the two institutions has been an essential ingredient of the partnership’s success. Initially, support and buy-in from institutional and senior leadership from both institutions were essential in developing the administrative and programmatic elements of the collaboration. As partnerships between individual investigators from Sri Lanka and the US blossomed, these new relationships became most important in sustaining the collaboration. Finding the right people and right research projects that met an important need locally were ultimately the most important ingredients to the success of the collaboration. The trust, rapport, and respect between the members of institutional teams and mutual appreciation of the benefits of partnership have been key elements for success. The importance of having dedicated champions and true advocates for the partnership within the two institutions also cannot be stressed enough. The local staff members who managed the administrative and scientific aspects of the collaboration were the third essential component for success.

### ‘Business model’

Funding is essential to initiating and sustaining collaborative partnerships. The collaboration started with a ‘shoestring’ budget consisting of internal funds from the institutions and assigned fieldwork funds received by graduate students for conducting their thesis projects; these funds were mostly used towards the 10-week fieldwork activities. Even with this relatively modest investment, the collaboration became productive, with much of the projects initially being driven by senior research students.

With demonstration of continued productivity, external funding from US sources such as the National Institutes of Health has also been secured. Similarly, Sri Lankan investigators were successful in securing funds through local sources such as the Sri Lanka National Science Foundation. The competition for these local funds is intense, and it took several years before enough mutual trust and enough preliminary joint work made this possible.

Successful local national grant funding is an important accomplishment and achievement for the program. It is not unusual in global health that the higher-resourced partner generally procures and manages funds, resulting in an unfortunate and inequitable power balance [82]. Diversifying to include funding from local sources, led by local investigators, as well as having students be the drivers and managers for the funds for their fieldwork projects, has helped mitigate this imbalance in our collaboration.

### Development of local laboratory

The collaboration was structured such that emerging objectives and priorities could be continually identified through regular joint meetings between investigators, stakeholders, and designated project leads at both institutions. One such emerging priority which was identified was increasing technical laboratory capacity at Ruhuna, essential for expanding the breadth and type of research that could be conducted on site and reducing dependency on testing overseas. Specific actions were taken to strengthen laboratory capacity, including the procurement of essential equipment, training of local personnel, and remote mentoring by Duke laboratory experts. The collaboration helped develop capacity on site such that Polymerase Chain Reaction (PCR) and immunoassays could be conducted, which proved valuable during the COVID-19 pandemic, when the Ministry of Health was assisted with surveillance. The laboratory has also helped support molecular testing for many other ongoing research studies.

### Students

Students have been enthusiastic and energetic catalysts for the research program, with Duke graduates and medical trainees dedicating extensive time in planning research projects and fieldwork. Both Ruhuna and Duke faculty emphasize publishing in global health journals, resulting in a strong record of peer-reviewed articles. While most students are from Duke due to Sri Lankan students’ schedule constraints, efforts are underway to expand Sri Lankan participation through research courses and master’s programs. The collaboration has also served as a transformative platform for students/research assistants from both institutions. Ruhuna students gained early exposure to global research standards, developing towards confident researchers/clinicians on public health. Duke students acquired cultural humility and insight into health differences through widespread fieldwork. Emphasizing fairness, shared authorship, and cross-cultural mentorship, the program fostered professional identities in collaboration, ethics, and global health leadership.

### Bidirectionality and twinning within different academic structures

Bidirectionality and mutual benefit are the ultimate goals for most global health partnerships, including the Ruhuna-Duke collaboration which initially favored Duke trainees, but is increasingly balanced through ‘twinning’ programs. Limited funding has restricted extended exchanges, yet it has been possible to bring investigators and trainees from Sri Lanka to Duke for both shorter and longer research and educational opportunities. The most active Ruhuna investigators have also been given secondary academic positions at Duke, and similarly, the faculty liaison on the Duke side has recently been appointed as Ruhuna faculty.

Furthermore, constraints on Sri Lanka’s clinical and academic systems (such as lack of research time and differing promotion metrics) challenge deeper research involvement for Ruhuna faculty, especially those with heavy clinical duties. It was important to understand these divergent metrics early, so that the needs of all partners could be taken into consideration and met.

## Future directions

### Research

As the collaboration continues to grow, the intention is to maintain the breadth of research activities in community medicine, occupational and mental health, and infectious diseases, while also venturing into related pressing public health areas such as noncommunicable diseases and environmental health under the One Health umbrella. The plan includes engaging more academic partners to help widen the mission, conducting interventional clinical trials that can directly answer clinical questions, and translating the findings to action and policy with increased involvement with the Ministry of Health. The local senior Sri Lankan ‘alumni’ of our collaboration, now placed in senior positions in the health system, help catalyze this work. Additionally, developing a more advanced molecular diagnostic laboratory at Ruhuna is planned, which could serve as a regional surveillance center for the Southern Province, performing testing real-time, and sharing data with the Ministry of Health to assist with outbreak detection and monitor changes in disease epidemiology.

### Collaboration within the country and regionally

The partnership has a longstanding history of collaborating with other entities in Sri Lanka and aims to expand and strengthen these links such as with SingHealth Duke-NUS Global Health Institute (SDGHI) and their partnership with the University of Jaffna in northern Sri Lanka, creating the potential for growing locally as well as regionally in Asia (Singapore, Vietnam, Indonesia, Philippines, and Thailand). The Sri Lanka North–South link provides an important opportunity for capacity building in the civil war-affected North and to explore epidemiology and clinical questions across contrasting historical, climatic, cultural, and geographical contexts.

### Improving training capacity

One of our key goals is to identify and support more Sri Lankan trainees and junior faculty through research training opportunities, taking as a role model the recently completed PhD at Ruhuna. Skills development of the trainees was monitored and supported through structured activities such as hands-on training, supervised fieldwork, regular monitoring sessions, and collaborative manuscript writing. Feedback from mentors and research participants, successful data collection, analysis, and not least authorship of peer-reviewed publications serve as good indicators of skill acquisition. We also aim to help address the exodus of trained health professionals and academics from Sri Lanka and help junior faculty obtain the requisite skills to apply competitively for independent funding and manage the program, while fostering more equal partnerships. We also plan to implement more formal evaluation tools to systematically assess the research skills development of these trainees.

### Infrastructure development

To facilitate the development of research and training capacity, which will both be greatly enhanced by shifting laboratory testing to the local setting, the collaboration will be investing in physical infrastructure to develop a new, state-of-the-art molecular diagnostic laboratory. This expansion within the Faculty of Medicine, University of Ruhuna, will include developing physical infrastructure available for delivering coursework and for administrative activities by the Duke-Ruhuna staff and faculty. This plan is being supported by strategic funds allocated by the Duke Hubert-Yeargan Center for Global Health and the Duke Global Health Institute and represents a major milestone in our collaboration’s progress.

### Key challenges

Over the past two decades, the collaboration encountered numerous challenges including limited infrastructure, administrative and financial barriers within the universities, difficulty in sustaining capacity-building efforts due to the short-term, contact-based nature of the supportive research staff, coordination/communication barriers across the institutions, ensuring equitable authorship recognition and distribution, and navigating cultural differences between the countries.

While the collaboration has made continuous efforts to strengthen the link between the Ministry of Health in order to foster a joint research culture and thereby contribute to policymaking and implementation, it has only recently experienced some success in this regard. Additionally, the partnership has pursued establishing a permanent category of research support staff within the university, but a formal structure for such positions has not yet been successfully implemented.

Several ethical dilemmas also emerged during different phases of implementation, primarily related to cultural barriers, trust-building in the context of international partnerships, and equitable authorship practices. Despite these obstacles, the partnership has progressed steadily, mainly due to the strong commitment, mutual support, and collaborative efforts of the teams from both universities.

## Conclusion

The breadth of educational and research activities and the strength of the relationship between partners in Sri Lanka and the US have helped sustain the Ruhuna-Duke academic collaboration over two decades, overcoming the many challenges encountered: the Tsunami, a brutal civil war, the Easter Bombings, COVID-19, as well as the recent government insurrection and severe economic crisis. The collaboration owes its success to the commitment, initiative, and dedication from faculty, students, and staff, and not least to the transparency and mutual trust that has increasingly been built and established.

## Data Availability

There are no participant data associated with this manuscript.
